# High Temperature, High Power Piezoelectric Composite Transducers

**DOI:** 10.3390/s140814526

**Published:** 2014-08-08

**Authors:** Hyeong Jae Lee, Shujun Zhang, Yoseph Bar-Cohen, StewarT. Sherrit

**Affiliations:** 1 Jet Propulsion Laboratory, California Institute of Technology, Pasadena, CA 91109, USA; E-Mails: yoseph.bar-cohen@jpl.nasa.gov (Y.B.-C.); stewart.sherrit@jpl.nasa.gov (S.S.); 2 Material Research Institute, Pennsylvania State University, University Park, PA 16802, USA

**Keywords:** piezocomposites, high temperature, high power, transducer, sensor

## Abstract

Piezoelectric composites are a class of functional materials consisting of piezoelectric active materials and non-piezoelectric passive polymers, mechanically attached together to form different connectivities. These composites have several advantages compared to conventional piezoelectric ceramics and polymers, including improved electromechanical properties, mechanical flexibility and the ability to tailor properties by using several different connectivity patterns. These advantages have led to the improvement of overall transducer performance, such as transducer sensitivity and bandwidth, resulting in rapid implementation of piezoelectric composites in medical imaging ultrasounds and other acoustic transducers. Recently, new piezoelectric composite transducers have been developed with optimized composite components that have improved thermal stability and mechanical quality factors, making them promising candidates for high temperature, high power transducer applications, such as therapeutic ultrasound, high power ultrasonic wirebonding, high temperature non-destructive testing, and downhole energy harvesting. This paper will present recent developments of piezoelectric composite technology for high temperature and high power applications. The concerns and limitations of using piezoelectric composites will also be discussed, and the expected future research directions will be outlined.

## 1. Introduction

Piezoelectric materials convert electrical energy into mechanical/vibrational energy and *vice versa*. Using the direct and converse piezoelectric effects, piezoelectric materials have been implemented for a wide range of sensors, actuators, and transducer applications. Within the past few years, the use of high-power and high temperature piezoelectric materials has grown and expanded significantly. A variety of applications exist where actuators, sensors and transducers able to operate at elevated temperatures (>100 °C) or high power condition would be extremely beneficial. These include non-destructive testing (NDT), structural health monitoring, energy harvesting, underwater acoustics, medical therapy and wire-bonding, to name but a few.

A wide diversity of piezoelectric applications has been the driving force for the development of an extensive range of piezoelectric materials. Most of the applications require a combination of properties, which generally necessitates a trade-off in other properties. Piezoelectric transducers for ultrasonic imaging, for example, may require high electromechanical coupling factors for broader bandwidth, low density for low acoustic impedance and mechanical flexibility. One single piezoelectric material phase does not provide all of these features, and the performance of ultrasound transducers is limited by the trade-off between high piezoelectric activity and low density with mechanical flexibility. Piezoelectric composites offer one tailored solution for this problem. As with low density polymer, the acoustic impedance of ferroelectric materials can be reduced, alleviating the acoustic impedance matching problems. Several electrical properties of the composites can be tailored to specific device requirements by varying the volume fraction of ferroelectric materials, offering a new range of material properties. The superiority of composites over monolithic piezoelectrics has been well recognized, being implemented in various transducer applications, such as non-destructive testing and medical diagnostics [1–4].

The uses of conventional piezocomposites have been limited in high power and/or high temperature applications due to their inherently low mechanical quality factors, relatively low thermal conductivity and high thermal expansion of the polymer fillers. A low mechanical quality factor causes power loss and internal heating under high power operations at resonance, while high thermal expansion coefficient of polymer filler results in cracking and de-bonding in the composite material, leading to structural failure at high temperatures. Meanwhile, a low thermal conductivity of polymer reduces thermal dissipation to surrounding environment and also results in localized hot spots adjacent to piezoelectric pillars in 1–3 composites that can melt the polymer. To overcome these issues, appropriate selection of composite components is of paramount importance, and this paper reviews the recent developments in high temperature and high power piezocomposites.

A brief outline of this paper is as follows: Section 2 reviews high temperature, high power piezoelectric materials and polymer materials that are available for piezocomposites and the effect of filler materials on the properties of piezocomposites is discussed. Section 3 presents the general concept of Figure of Merit (FOM) and the importance of connectivity of piezo/polymer composites and relevant design criteria to fabricate the composites. Also, this section presents analytical methods predicting effective material properties of piezocomposites and available composite fabrication methods. Section 4 discusses the potential of composite transducers for various high power applications including acoustic radiation force impulse (ARFI) imaging and ultrasound therapy and ultrasonic wire-bonding, as well as high temperature applications including non-destructive testing and energy harvesting. Advantages of piezocomposites over monolithic counterparts for these electromechanical applications will be discussed. Finally, a brief summary and future direction are presented in Section 5.

## 2. Composite Materials

### 2.1. Piezoelectric Materials

A useful guideline for the selection of the appropriate piezoelectric materials is figure-of-merit (FOM), which depends on the applications. For the case of transducer, sensor and energy harvester, for example, the FOM is the product of piezoelectric charge and voltage coefficients (d_ij_.g_ij_), where higher value of these coefficients gives higher electromechanical coupling that is defined as the ratio of stored electrical energy to applied mechanical energy or vice versa. The FOM for high power applications operating at their resonant frequencies is the product of the piezoelectric strain coefficient and mechanical quality factor (d_ij_.Q_m_), which is proportionally related to the vibration velocity and output power. Note that time-averaged power dissipation per unit volume of piezoelectric materials is proportional to the dielectric and mechanical loss factors, where mechanical loss tends to be the major source of power dissipation at resonance, while dielectric loss is the major contributor at off resonance. In general, the materials with high mechanical quality factor (low mechanical loss) possess low dielectric loss; thus, mechanical quality factor is a particularly important parameter for high power applications [5–7].

For specific high temperature applications, phase transition at elevated temperature is an important consideration along with FOM. This is because at a certain temperature, the material transforms from a ferroelectric phase to a high symmetry non-ferroelectric phase, whose transition temperature is referred to as the Curie temperature. Above this temperature, the material is permanently depolarized and cannot be used for piezoelectric applications. Therefore, the maximum operating temperatures of ferroelectric materials are determined by their respective Curie temperatures. In practice, the operating temperature must be substantially lower than the Curie temperature in order to minimize thermal aging and property degradation. As a general rule of thumb, it is not recommended to exceed half of the Curie temperature of ferroelectric materials for extended periods.

There is a large number of piezoelectric materials that could be candidates as high temperature piezoelectrics, including polycrystalline ceramics with perovskite, tungsten bronze, bismuth layer (BLSF-Aurivillius) or double perovskite layer (PLS) structures. However, the number of available high temperature piezoelectric materials is very limited when doing a trade-off between the Curie temperature and piezoelectric properties, where materials possessing high T_C_ generally exhibit lower piezoelectric sensitivity [8,9]. Polycrystalline ferroelectric ceramics, represented by PZT, have been the material of choice for various piezoelectric applications owing to their high piezoelectric and electromechanical properties. With additives of donors or acceptors, PZT can be modified to produce a soft-type or hard-type PZT to meet specific device requirements; for example, soft-type PZTs (PZT5H and PZT5A) are suitable for transducer, sensor, and actuator, while hard-type PZTs (PZT4 and PZT8) are suitable for resonance-type actuators (ultrasonic motor) and high power applications that drive the transducer at their resonant frequencies.

Table 1 lists some of the piezoelectric materials that can be used for high temperature and/or high power piezoelectric applications. In general, perovskite piezoelectric materials offer high dielectric and piezoelectric properties, but with relatively low Curie temperatures compared to non-perovskite piezoelectric materials [8]. Lead-free piezoelectric materials with perovskite structure, such as (K,Na)NbO_3_ (KNN) and (Bi,Na)TiO_3_ (BNT) based ceramics, generally have inferior dielectric and piezoelectric properties as well as lower phase transition temperatures, in comparison with lead-based piezoelectric materials. However, hard lead free piezoelectric materials offer high mechanical quality factor and low dielectric loss, comparable to lead-based piezoelectric materials [8–10]. In addition, it was reported that BNT-based ceramics showed minimal variation of resonance frequency shift and mechanical quality factor under hard drive condition and high level vibrations, promising for high power piezoelectric applications [11]. For the case of acceptor (Mn) modified relaxor-PT single crystals, such as Pb(In_1/2_Nb_1/2_)O_3_-Pb(Mg_1/3_Nb_2/3_)O_3_-PbTiO_3_ (PIN-PMN-PT), they possess very high piezoelectric strain coefficient (>1000 pm/V) and mechanical quality factor (>800), offering higher FOM (d_ij_.Q_m_) compared to polycrystalline counterparts; however, their usage temperature range is limited by a low ferroelectric—ferroelectric phase transition temperature (∼123 °C) [12].

It is clear that at present a trade-off must be made in selecting piezoelectric materials for high temperature or high power applications. A more detailed discussion on the high temperature and high power piezoelectric materials can be found elsewhere [13–16].

### 2.2. Polymer Materials

Selection of passive polymer materials is as important as the selection of piezoelectric materials for composites, especially for high power and/or high temperature applications. It has been reported that the presence of polymers is a primary limiting factor for use of piezocomposites in harsh environmental conditions [26]. A wide variety of polymers has been used as the passive components in piezocomposites. The reported properties of some of the polymer materials are compiled and listed in Table 2. Elastic modulus (c_ij_) and wave velocities (*v*) of the polymers can be derived from the following equations assuming that the properties of polymer materials are isotropic:

(1) $$c_{44}=\frac{Y}{2(\sigma +1)},c_{12}=\frac{2c_{44}\sigma }{1-2\sigma },c_{11}=c_{12}+2c_{44}$$

(2) $$\nu_{L}=\sqrt{\frac{c_{11}}{\rho },}\nu_{s}=\sqrt{\frac{c_{44}}{\rho }}$$

In general, low Young's modulus (Y) and low Poisson's ratio (σ), *i.e.*, soft polymers, are preferred for use as the passive materials since they provide less restriction on the piezoelectric vibrations, and this translates into a large electromechanical coupling piezocomposites. A detailed discussion of the effects of Young's modulus and Poisson's ratio on composite performance was given by Smith [37]. The drawbacks of soft polymers are an increase in machining/polishing and fabrication difficulties, as well as the inability to withstand external pressure characteristics of large ocean depth and downhole applications.

High thermal conductivity (k_c_) is another important consideration for high power and/or high temperature applications. In general, the thermal conductivity of polymer materials is relatively low and it is on the order of 0.2–0.3 W/mK. This hinders heat dissipation into the surrounding medium, as the heat is trapped in the polymer matrix, limiting their use in high duty cycle, high drive conditions. High thermal conductivities of polymers can provide a thermal pathway, enhancing thermal management within composites [30,38,39]. In addition, high thermal expansion coefficient of polymers compared to that of piezoelectric materials is another limiting factor for the piezocomposites at elevated temperatures. For example, the thermal expansion coefficient of PZT piezoelectric materials is approximately 2–3 ppm/°C, whereas that of the polymer is 60–70 ppm/°C, which is dramatically increased when the temperature is close to its glass transition temperature. To mitigate low thermal conductivity and high thermal expansion coefficient of polymers, the appropriate filler materials can be added to the resin prior to polymerization, such as thermally conductive particles or nonelectrically conductive inorganic filler materials. However, the drawback of this approach is that such fillers can cause a dramatic increase in elastic stiffness and loss of the polymer materials, deteriorating the composite transducer performance [40–43]. In addition, the incorporation of such filler particles increases the viscosity in the uncured state, which makes it difficult for filling in the kerf during composite fabrication.

## 3. Piezocomposite Design and Fabrication

### 3.1. Composite Design

The properties of a composite are strongly associated with the connectivity of its components. Connectivity is defined as the arrangement of the active and passive phases in the composite. The concept of connectivity is a convenient way to describe the manner in which the individual phases are self-connected (continuous). For piezoelectric composites, various different connectivities have been studied including: 0-3, 1-3, 2-2, 2-3, 3-0, 3-1, 3-2, and 3-3, where the first number in the notation represents the connectivity of the electromechanically active phase, and the second number refers to the connectivity of the electromechanically passive phase. The “0” means that material is in the form of particles. Composites with 0-3 connectivity, for example, are composed of randomly dispersed piezoelectric particles, such as PZT or PbTiO_3_, in the 3D polymer phases, and this connectivity has been investigated for sensor applications. The advantages of this connectivity are high flexibility with relatively high piezoelectric voltage coefficient, which allows for the fabrication of more complicated shapes than other forms of composites. 3-3 connectivity can be regarded as 0-3 composites with a high concentration of piezoelectric particles, offering higher piezoelectric activities compared to 0-3 composites. 2-2 connectivity consists of alternating layers of the two phases, and this pattern has been used for various sensor, transducer and actuator applications, such as multilayer actuators, bimorph actuators or sensors, piezoelectric stack actuators and medical linear array transducers [44–46].

**Figure 1.**
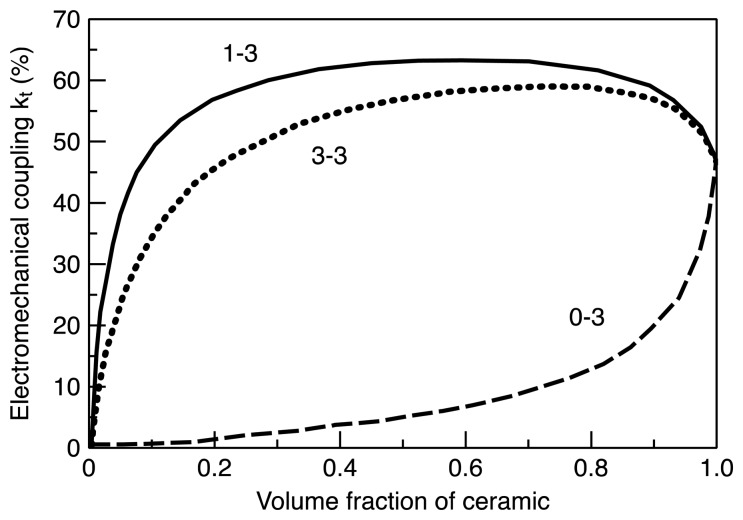
Electromechanical coupling factors of 1-3, 3-3 and 0-3 piezocomposites as a function of volume fraction of piezoelectric ceramic (Reprinted with permission from [45]. © [1998] IEEE.).

**Figure 2.**
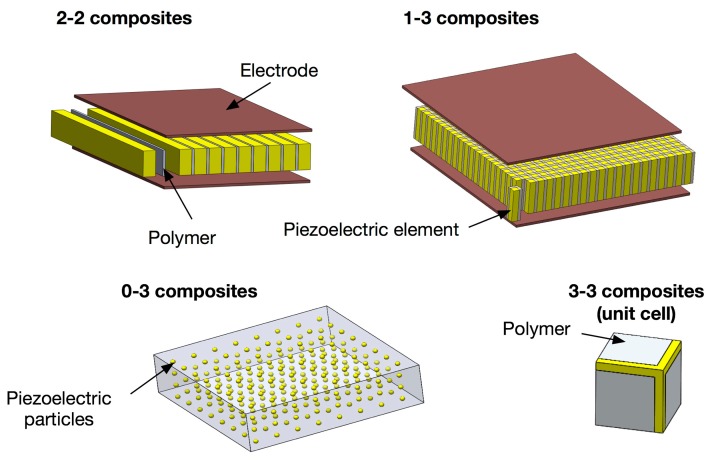
Schematic representations of piezocomposites with 2-2, 1-3, 0-3 and 3-3 connectivities.

The most widely used composite connectivity is 1-3 connectivity since this connectivity can effectively utilize the geometrical advantages of piezoelectric materials, offering highest electromechanical coupling factors. Example of electromechanical property depending on connectivity and volume fraction is given in Figure 1. Because of this merit, 1-3 composites have been used for a wide range of sensor and transducer applications, including medical imaging, NDT, and underwater acoustic transducers [44,47–52]. Schematics of the 2-2, 1-3, 0-3 and 3-3 connectivities are shown in Figure 2.

**Figure 3.**
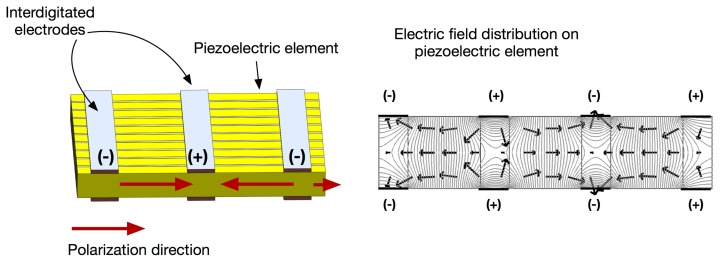
Schematic of fiber composite actuator (**Left**); Electric field distribution on piezoelectric element, where arrow indicates the direction of electric field (**Right**).

Piezoelectric Fiber Composites, such as Macro Fiber Composites (MFC) [53], followed by Active Fiber Composite (AFC) [54], are recently developed piezoelectric composites that offers light weight, robust construction and increased actuation strain energy density, in comparison with monolithic piezoelectrics as well as conventional piezocomposites. This type of composites has uniaxially aligned piezoceramic fibers surrounded by a polymer matrix, similar to 1-3 composites with electrodes on the side, but possess interdigitated electrodes that is produced using photolithography method. With an applied voltage, the interdigitated electrodes induce curved longitudinal electric fields along the length of each fiber, allowing for piezoelectric fibers in the d_33_-mode and offering nearly twice the strain actuation. The basic concepts of the interdigitated electrode piezocomposite are illustrated in Figure 3. The maximum peak-to-peak actuation strain is reported to be approximately 1500–2000 parts-per-million in the longitudinal direction under the applied voltage of 2 kV [55]. These advantages open a wide range of potential applications, including structural control applications, structural health monitoring and active damping of high-frequency vibration [55–59].

### 3.2. Analytical Model of Piezocomposites

Effective material parameters for piezocomposites, such as 2-2 or 1-3 connectivity, can be determined using analytical mixing rules based on the uniform field method under the plane stress assumption [37]. For a two-phase composite, there are two possible cases: series connection and parallel connection. The series connection consists of two phases that are laid in layers parallel to the electrode, while the parallel connection consists of two phases that are laid in layers perpendicular to the electrode. The applicable equations for the piezoelectric coefficients (neglecting transverse coupling) for series connection can be written as follows:

(3) $$\bar{d_{33}}=\frac{(1-\nu ){ɛ}_{33}^{T}d_{33,p}+d_{33}\nu {ɛ}_{11}}{(1-\nu ){ɛ}_{33}^{T}+\nu {ɛ}_{11}}$$

where v, d_33,p_ and ε_33_^T^ are volume fraction, piezoelectric strain coefficient and permittivity of piezoelectric phase, respectively. Piezoelectric strain coefficient and permittivity of polymer phase are represented by d_33_ and ε_11_, respectively. The equation indicates that for series connection, the piezoelectric strain coefficient is decreased with increasing polymer phase.

Interesting characteristics of piezocomposites can be found in the parallel connectivity. For composites with parallel connectivity, such as 1-3 composites, the stress acting on the more compliant phase will be transferred to the stiffer phase. Under these circumstances, the effective values for the dielectric permittivity and piezoelectric strain coefficients can be obtained using the following equations:

(4) $$\begin{array}{l} \bar{{ɛ}_{33}^{T}}=\nu \left[{ɛ}_{33}^{T}-d_{33}^{2}\frac{1-\nu }{\nu s_{11}+(1-\nu )s_{33}^{E}}\right]+(1-\nu ){ɛ}_{11}\\ \bar{d_{33}}=\frac{\nu d_{33}s_{11}+(1-\nu )d_{33,p}s_{33}^{E}}{\nu s_{11}+(1-\nu )s_{33}^{E}},\\ \bar{d_{31}}=\nu \left[d_{31}+\frac{d_{33}(1-\nu )(s_{12}-s_{13}^{E})}{\nu s_{11}+(1-\nu )s_{33}^{E}}\right] \end{array}$$

where s_ij_^E^ are s_ij_ are elastic compliance of piezoelectric and polymer phases, respectively. Piezoelectric voltage coefficient of the composites can be calculated using the following relation:

(5) $$\bar{g_{ij}}=\frac{\bar{d_{ij}}}{{ɛ}_{ii}^{T}}$$

Piezocomposites are particularly suitable for hydrostatic sensor applications, such as hydrophone. The hydrostatic piezoelectric coefficients, which are important parameters in assessing piezoelectric materials for use in hydrostatic applications, are related to the longitudinal and transverse coefficients through the following relationship: d_h_ = d_33_ + d_31_ + d_32_ and g_h_ = g_33_ + g_31_ + g_32_. For the case of PZT ceramics, a longitudinal coefficient is positive and transverse coefficients are negative, and both the transverse direction coefficients have magnitudes nearly equal to half those of d_33_ with opposite sign, *i.e.*, d_33_ ≈ −2 d_31_, resulting in the cancellation between coefficients. For composites, the negative part of the piezoelectric response can be reduced by the clamping from the polymer phase, which allows for a large improvement in hydrostatic responses at low volume fraction of piezoelectrics [60].

**Figure 4.**
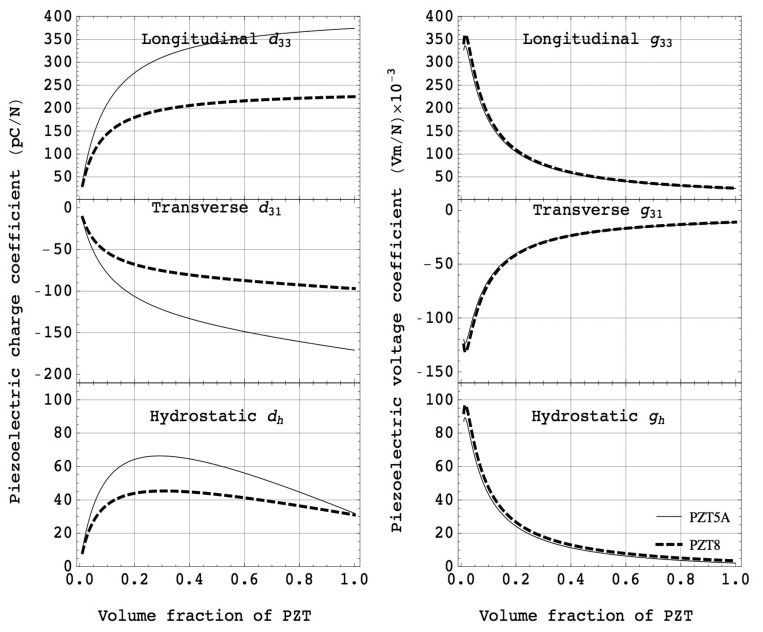
Computed piezoelectric charge, voltage and hydrostatic coefficients as a function of volume fraction for PZT5A (solid line) and PZT8 (dashed line).

Figure 4 shows examples of piezoelectric charge coefficients and voltage coefficients as a function of volume fraction of piezoelectric ceramics (PZT5A and PZT8). It can be observed that the PZT composites showed almost constant values of piezoelectric charge coefficient for a wide range of volume fraction.

This is because PZT ceramics have much higher elastic stiffness compared to polymers, *i.e.*, s_11_ > s_33_^E^. Thus, the piezoelectric strain coefficient of composites is approximately equal to that of piezoelectric ceramics down to the volume fraction of 0.5. On the contrary, the dielectric permittivity of composites is a linear relationship with volume fraction of piezoelectric elements due to much higher dielectric permittivity and lower elastic compliance for piezoelectric materials compared to those of polymers. The large piezoelectric effect together with a small dielectric permittivity gives the largest piezoelectric voltage coefficient in both the longitudinal and transverse modes, suitable for various sensors and energy harvesting applications.

**Figure 5.**
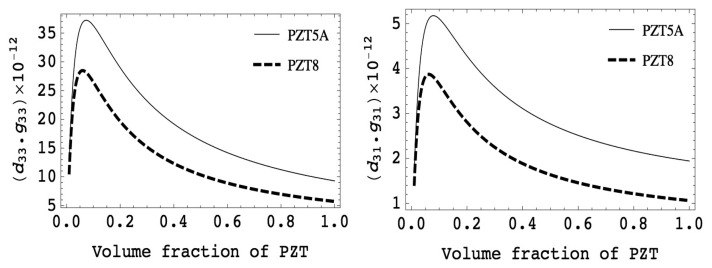
Computed figure-of-merits (d_33_·g_33_ and d_31_·g_31_) for transducers and sensors as a function of piezoelectric volume fraction for PZT5A and PZT8.

Figure 5 shows the volume fraction dependence of the product of piezoelectric charge and voltage coefficients, which is the figure of merit for sensor and/or energy harvesting applications. It can be seen that a great improvement appears at low volume fraction of piezoelectrics. The square of electromechanical coupling factor, defined as the ratio of the mechanical energy to the input electrical energy, can be computed using the Equation (6). Note that the electromechanical coupling is the product of piezoelectric charge and voltage coefficient divided by elastic compliance, where the elastic compliance of the composites is increased with decreasing volume fraction of piezoelectrics. This results in nearly constant coupling values over a wide range of volume fraction. These characteristics are particularly interesting for various ultrasonic transducer applications, such as medical imaging, high intensity focused ultrasound (HIFU), underwater acoustic, and NDT, as this enables broader bandwidth and higher sensitivity of transducers:

(6) $$k_{ij}^{2}=\frac{d_{ij}^{2}}{{ɛ}_{ii}^{T}s_{jj}^{E}}=\frac{d_{ij}\cdot g_{ij}}{s_{jj}^{E}}$$

### 3.3. Fabrication Methods

Various manufacturing techniques have been reported and used to produce piezocomposites, including rod placement technique, dice-fill technique, ultrasonic cutting, injection molding, lost mold, laser machining, co-extrusion, tape lamination and fiber insertion methods. Detailed reviews of several composite fabrication techniques can be found in [61,62]. Among these fabrication methods, the fiber insertion technique, injection molding, dice and fill are the most popular methods for the fabrication of piezocomposites, which will be reviewed briefly in the following paragraphs.

The rod placement technique, also called “pick and place”, was the earliest technique, developed and patented by Klicker *et al.* [60,63], whereby sintered ceramic rods are picked and placed in a polymer matrix to fabricate 1-3 composites. The ceramic rods can be formed by extrusion, followed by firing. The limitation of this method is that the process is time-consuming as the extruded rods become finer.

**Figure 6.**
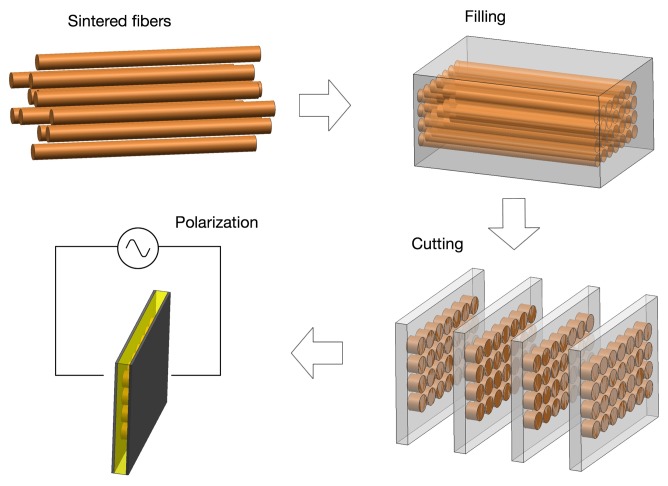
Process chart of 1-3 fiber composite fabrication process.

The fiber technique was introduced to overcome the difficulties of the early stage of the pick and place technique. The motivation for the development of piezoelectric fibers first arose from the need for fine scale fibers associated with high frequency medical imaging applications. Fine scale fibers have been prepared via viscous suspension spinning process (VSSP) [64,65] or sol-gel techniques [66–68], where the use of fiber diameters as small as 50 and 10 microns were reported, enabling up to 70 MHz frequency ultrasound transducers. The advantage of this method, besides the fine scale fibers, is the ability to randomly distribute the piezoelectric elements, which can contribute to the suppression of spurious modes caused by Lamb waves. This fiber processing technique has been developed and implemented by Smart Material Corp. (Sarasota, FL), where the epoxy infiltration of fiber bundles are aligned regularly or randomly, followed by curing and slicing to a desired thickness [69]. The process and product are shown in Figure 6.

**Figure 7.**
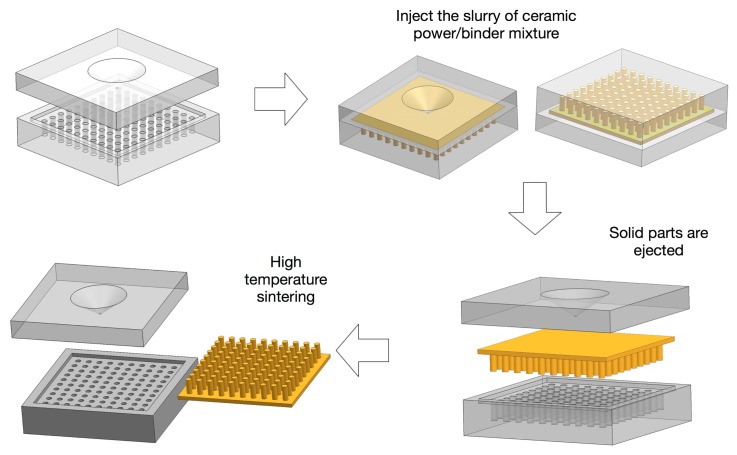
1-3 piezocomposite fabrication using the injection molding technique.

For injection molding technique, the slurry of ceramic powder/binder mixture is injected into the molds, then a net shape 1-3 or 2-2 preform is ejected from a metallic mold. The preforms are then heat treated to remove the binder, followed by sintering. The final piezocomposites are made by backfilling the structure with a desired polymer, followed by back-grinding the material until all of the piezoelectric posts are exposed. The process is schematically shown in Figure 7. The advantage of this technique is that the fabrication method is simple and fast, thus, it is suited for mass production of composites with identical design. The lost mold technique process is similar to the injection molding technique, except that a sacrificial plastic mold is used instead of a metallic mold, which is removed during binder burn-out process. The limitation of both methods arises from the mold; the design and fabrication of molds are expensive and time consuming. Thus, this method is not suitable for the design verification and performance optimization stages of piezocomposites. Note that to overcome this limitation, it is possible to use a soft-molding technique that allows the mold to be removed by peeling it off so it can be reused [47,70–72].

**Figure 8.**
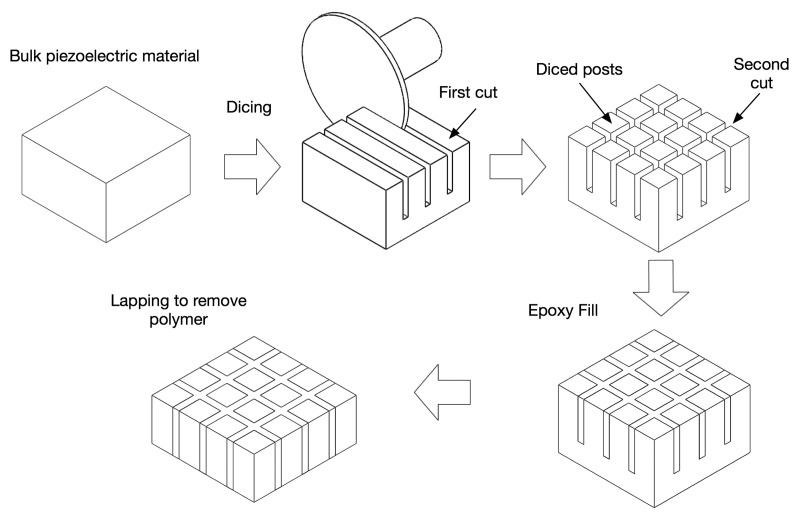
1-3 composite fabrication using the dice-and-fill technique.

The dice and fill technique is currently the most widely accepted method for the fabrication of piezocomposites due to its simple process and design flexibility. This method was first reported by Savakus *et al.* [56]. The dice and fill method involves making a series of parallel cuts on a piece of bulk piezoelectric material with a mechanical dicing saw. The material is then diced in the perpendicular direction to produce posts with a rectangular cross section. The diced material is backfilled with a polymer, then the base ceramic support is removed by polishing (see Figure 8). Note that Macro Fiber Composites (MFC) are also fabricated using the dice-fill method, where piezoelectric fiber sheets are machined from monolithic wafers of piezoceramic material using a dicing saw.

One of the limitations of the dice-fill technique is inability to fabricate high frequency composites (>20 MHz). In particular, the microstructure of ceramics has a strong impact on the quality of final composite structure. For example, as ultrasound transducers become smaller with increasing frequency, there is increasing awareness of the effects of surface damage introduced during composite machining because the damage layer volume increases in relation to the active piezoelectric materials. The use of pore free and fine grain ceramics improves the machinability of piezocomposite structure [73].

For the fabrication of piezocomposites with an operational frequency range above 20 MHz, a photolithography based micromachining process was developed by TRS in order to fabricate high frequency ultrasound transducers based on relaxor ferroelectric single crystals [74–81]. The advantage offered by this technique lies on the ability to realize narrow channels or kerfs less than 10 microns, enabling high aspect ratio of piezoelectric elements. In addition, lithography based micromachining technology uses dry etching method, which results in less surface damage on the piezoelectric elements compared to the composites with conventional dicing-fill method, suitable for the fabrication of high frequency piezocomposite transducers.

## 4. Potential Applications

### 4.1. Medical Imaging and Therapeutic Ultrasound

Piezoelectric transducers have been widely used in a range of medical applications, including ultrasound diagnostic imaging, acoustic radiation force impulse (ARFI) imaging, and ultrasound therapy, such as high intensity focused ultrasound (HIFU). Among them, ultrasound diagnostic imaging is one of key examples for the successful application of piezoelectric composite technology. The main benefit of a piezocomposites is an enhanced electromechanical coupling as a result of the optimum geometry and aspect ratio of piezoelectric elements in composites. High coupling factors contribute to the broadening of the bandwidth and increase in energy transfer, resulting in a great improvement of the signal/noise ratio. A low acoustic impedance of piezocomposites allows for a good energy transfer between transducers and propagating medium, such as water or tissue, as a result of better impedance matching. In addition, piezocomposite materials can be mechanically shaped, allowing for the fabrication of transducers with concave or convex surface due to the flexibility of the polymer phase. The advantages of composites over monolithic piezoelectrics for medical transducer applications are summarized in Table 3 [51,52].

In contrast to medical diagnostic imaging, ARFI imaging utilizes brief, high intensity pulses to measure the magnitude of displacement of tissue with high contrast, which allows for evaluating the overall health of the tissue. Thus, it would be advantageous when the transducer can deliver high transmit power without increasing its face temperature or harming the tissues. For the case of therapeutic HIFU, the required transmit power and intensity levels are much higher than ARFI imaging to deliver focused, high intensity ultrasonic energy to the target area for the thermal ablation of malignant and benign tumors [82–87]. The inherently low mechanical quality factor of piezocomposites, however, limits their usage for these applications due to heat generation associated with high power operations. Furthermore, the polymer phase generally exhibits a low thermal conductivity (0.2 ∼ 0.3 W/mK), hindering heat dissipation into surrounding medium.

There have been attempts to improve mechanical quality factor of piezocomposites by selection of optimized composite components. Approaches to improve high power characteristics of piezcomposites are to use an appropriate passive material, such as low elastic loss or high thermal conductivity polymer, combined with a low loss piezoelectric material. It was shown that the incorporation of high thermal conductivity (>1 W/mK) polymers resulted in a significant reduction in the mechanical quality factor of piezocomposites, being less than 100, though they offer a better thermal management for the composites.

A low mechanical quality factor of the piezocomposites is due to the fact that high thermal conductivity polymers generally have relatively high values of elastic modulus, resulting in a high structural damping. In contrast, the composites filled with polymers that have low values of elastic modulus and losses offered relatively high mechanical quality factors, being >380–400, and consequently higher dynamic strains with respect to drive field under 50 °C isothermal condition [11,28,88], indicating that this kind of piezocomposites requires smaller input power to achieve the same level of output power compared to low mechanical quality factor counterparts, resulting in smaller heat generation, potentially overcoming the shortcoming of conventional piezocomposites.

**Figure 9.**
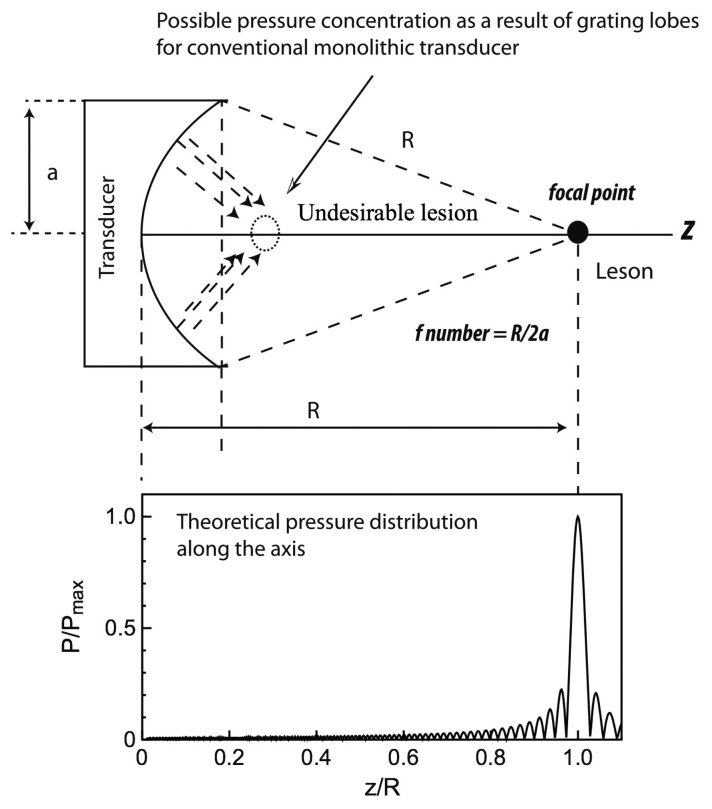
Theoretical on-axis normalized axial pressure profile from spherically focused ultrasound transducers whose radius of curvature is R.

Another advantage of piezocomposites is a reduced lateral vibration mode of piezoelectric transducers. Conventional monolithic piezoelectric transducers suffer from a large lateral vibration mode, which leads to grating lobes as a result of surface wave propagating from the edges of transducers. This leads to safety issues owing to high energy focusing or a formation of lesions on the unwanted regions. It was reported that the use of 1-3 piezocomposites could reduce this effect significantly [82], providing almost theoretical pressure distribution along the axis, as schematically depicted in Figure 9.

### 4.2. High Power Ultrasonic Wire-Bonding

Ultrasonic wire bonding has been used extensively in making electrical connections in microelectronic packaging. Ultrasonic wire-bonding transducers consist of a stack of hard type piezoelectric ring transducers that is maintained in compression between a backing and a horn by a pre-stress bolt. The presence of the horn amplifies the displacement induced by the piezoelectric stack when it is driven at its fundamental half-wavelength axial mode. The piezoelectric stack, where a number of alternately poled piezoelectric layers are connected mechanically in series and electrically in parallel, gives the increased piezoelectric strain coefficient that is proportional to the number of the piezoelectric layers. This kind of device is referred to as a Langevin type transducer, and they have been implemented for various transducer and actuator applications, such as Tonpilz transducers, ultrasonic drilling, and ultrasonic motors [89–91].

**Figure 10.**
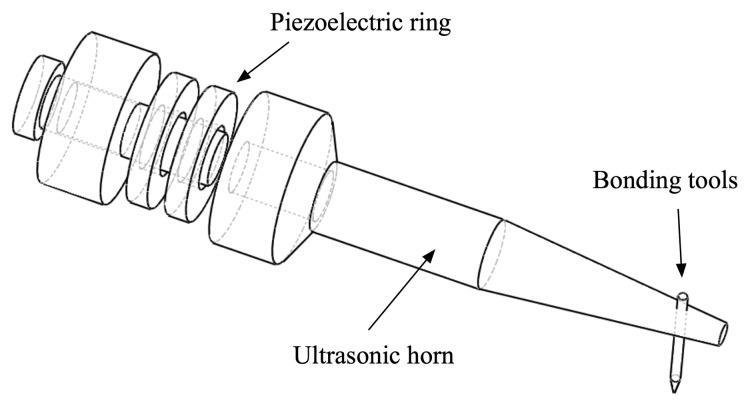
Schematic representation of the ultrasonic wire-bonding device.

Traditionally, the operating frequency of ultrasonic wire-bonding is ∼60 kHz, but higher frequency (100–150 kHz) wire-bonding have been investigated by several authors due to the advantages of better welding at lower temperatures in shorter bonding times [92,93]. Example of schematic representation of ultrasonic wire-bonding is shown in Figure 10, exhibiting a conventional Langevin type arrangement. Note that the axial length of piezoelectric ring transducers is comparable to the diameter of the ring. This results in mode coupling of radial (f_R_) and/or wall-thickness (f_W_) resonance to the desired axial mode of the transducer (f_T_), where resonant frequencies of each mode can be calculated using following equations:

(7) $$f_{T}=\frac{1}{2L\sqrt{\rho s_{33}^{E}}},f_{R}=\frac{1}{\pi {\text{D}}_{1}\sqrt{\rho s_{11}^{E}}},f_{W}=\frac{1}{2{\text{D}}_{2}\sqrt{\rho s_{11}^{E}}}$$

where subscripts T, R, and W represent thickness, radial and wall thickness, respectively. D_1_ and D_2_ are outer and inner diameter, respectively. It should be noted that when the non-axial mode vibrations are coupled to the axial mode vibration, the purity of axial motion is reduced, and the bond quality, such as bond width, is deteriorated due to the effect of lateral vibrations. In addition, monolithic piezoelectric materials, such as PZTs, have large radial and lateral mode vibrations, resulting in combined axial and nonaxial vibrations during operations owing to the mode coupling effect. 1-3 piezocomposites can alleviate this issue, as this structure reduces the lateral vibration amplitudes of piezoelectric elements. This allows for the suppression of the mode coupling effect and to maintain a pure axial mode, consequently improving overall bond quality.

Another advantage of the use of piezocomposite in ultrasonic wire-bonding is its reduced mechanical quality factor of an ultrasonic horn that generally have very high mechanical quality factor >1000. In general, a high mechanical quality factor is desirable for most high power applications, offering increased dynamic strain and reduced heat generation at resonance at the expense of operation bandwidth. For the case of wire-bonding applications, however, high mechanical quality factor limits operational bandwidth, leading to the difficulties in load changes and frequency tuning. Also, it causes a longer bonding time because high mechanical quality factor resonators need a longer time to reach the equilibrium state. It was reported that the piezocomposite transducer effectively reduces the rise and fall time of the vibration during wire bonding process, benefiting high-speed bonding machine [93,94].

### 4.3. High Temperature Non Destructive Testing

Ultrasonic techniques for nondestructive testing (NDT) or non-destructive evaluation (NDE) have been widely used in industry, mechanical engineering, civil engineering, and aeronautical engineering, where the pulse transit time method is used to monitor the material properties and integrity of critical structures. Although most NDT or NDE methods are applied at room temperature, there is a growing interest in high-temperature ultrasonic transducers in the field of high temperature pipeline inspection and health monitoring on steam pipe [13,95–97].

The advantages of piezocomposites are apparent in NDT and NDE applications as they offer wide bandwidth and flexibility. The flexibility of piezocomposites is particularly useful to curved pipeline or steam pipe monitoring as pipe curvature can cause wave losses, greatly reducing the signal-to-noise ratio. However, the major challenge to operate NDT technologies in a high-temperature environment is material survivability. Conventional PZT/epoxy composites are limited in the temperature usage range of < 100 °C, due to the relatively low glass transition temperature (T_g_) and high thermal expansion coefficient (TEC) of the polymer fillers. Thus, internal stress induced by the different TEC of ceramics and polymers gives rise to cracking and debonding in the composites and leads to structural failure.

Recently, Li *et al.* [98] reported high temperature piezocomposites using high temperature piezoelectric materials including modified PZT5A (TRS203, TRS Technologies Inc., State College, PA, USA) and glass sphere modified epoxy fillers (Duralco 4703, Cotronics Corp., Brooklyn, NY, USA), where the Curie temperature of TRS203 (380 °C) is higher than that of conventional PZT5A ceramics (360 °C), meanwhile, glass spheres were used to reduce the thermal expansion coefficient of the polymer matrix. Note that although the difference of Curie temperature between PZT5A and modified PZT5A (TRS203) is not significant, TRS203 materials showed the improvements in thermal stability for high temperature applications operating below 250 °C compared to conventional PZT 5A [99,100].

**Figure 11.**
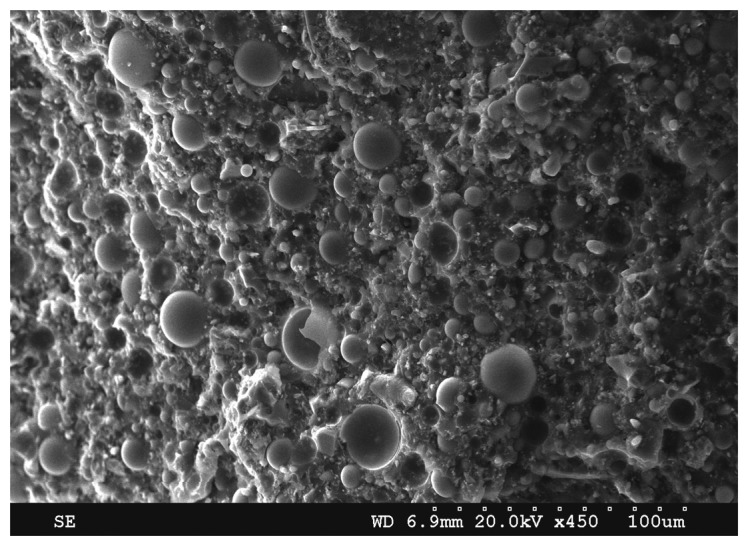
SEM cross-sections of a glass sphere modified epoxy.

An example image of a glass sphere modified epoxy is shown in Figure 11, where glass spheres are distributed in the polymer matrix. It was shown that the thermal expansion of the glass sphere modified polymer could be reduced from 68 ppm/°C to 59 ppm/°C at 250 °C when the glass sphere volume percent was increased from 0% to 12%. Above 12% of glass sphere volume percent, the thermal expansion of the polymer was further decreased; however, viscosity of the polymer was too high over 12% in volume, leading to an increase in the porosities in the polymer matrix. The results showed that the glass sphere (12%) modified composites showed no electrical and structural failure at 250 °C for up to 500 h with minimal electrical property variations, whereas PZT/epoxy (0%) composites showed cracks and the electrodes were damaged after thermal aging for 200 h due to the debonding of the ceramic pillar and polymer. Of particular interest is the electromechanical coupling factors of glass sphere (12%) modified composites maintained the similar values from room temperature up to 300 °C [97].

Similar investigations were conducted by the same research group [100], studying the temperature-dependent dielectric and electromechanical properties of 1-3 piezocomposites using new high Curie temperature (T_c_ ∼ 450 °C) piezoelectric materials, BiScO_3_-PbTiO_3_ (BS-PT), combined with high glass transition temperature epoxy material, cyanate ester, AroCy XU 371 (T_g_ ∼ 345 °C). The temperature coefficient of the dielectric permittivity was found to be less than 4% in the temperature range of 25–300 °C, with the dielectric loss being increased slightly from 1% to less than 5% at 300 °C. The electromechanical coupling factors were increased from 58% to 69% when the temperature increased from 25 to 300 °C, approaching the value of the monolithic BS-PT ceramics, due to the polymer softening. These results demonstrate the feasibility of 1-3 piezocomposites for use in various high temperature transducer applications at elevated temperature up to 300 °C.

### 4.4. High Temperature Energy Harvesting

Piezoelectric materials can be used for vibration based energy harvesting devices, which converts the random or generated vibrations present in the environment into useful amounts of electrical energy. An example that is required in an extreme environment is energy harvesting downhole in oil producing wells where the ambient pressures may reach to 30,000 psi and the temperature is up to 200 °C. Local power production is crucial since transmitting power from the surface is complicated by the difficulty of making electrical connections in a wet environment and across production packers. If the power can be produced locally by energy harvesting of the oil flow, the need to transmit power down the hole is removed, and downhole devices can be powered locally which reduces the overall complexity and difficulty of the system [101].

**Figure 12.**
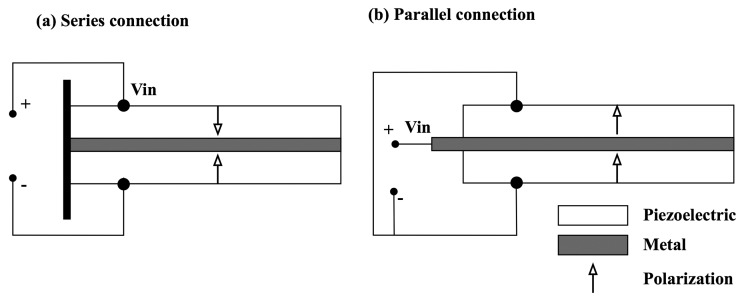
Schematic diagram of bimorph composites energy harvester with (**a**) series and (**b**) parallel connections.

Cantilever type piezoelectric layers, such as bimorphs, are typically used for energy harvesting devices, whose composites consist of two thin piezoelectric layers sandwiching a thin elastic metal layer. Two types of connections are generally used. One is the series connection, where the two piezoelectric layers have opposite polarization directions, the other is parallel connection, where the two piezoelectric layers have the same polarization directions. Schematic representations of the bimorph designs are illustrated in Figure 12. As shown, since the electric voltage is applied across the total thickness of the bimorph for the case of series connection, this configuration produces a larger voltage output, but a lower current output. In contrast, the electric voltage for parallel connection is applied between the intermediate electrode and the top/bottom electrodes, resulting in a larger current output, but a lower voltage output.

The constitutive equations describing the behavior of the cantilever type piezoelectric bimorphs were derived by Smits *et al.* [102–104] under static and dynamic conditions. If only an external force F is acting at the bimorph tip under an open circuit condition, generated electrical charge (Q) and voltage (V) can be obtained using the following equations:

(8) $$\begin{array}{c} Q\sim \frac{3d_{31}L^{2}}{4h^{2}}*F,\\ V_{\text{oc}}\sim \frac{3g_{31}L}{8wh}*F, \end{array}$$

where L, w, and h are length, width, and thickness of a piezoelectric element, respectively, F is mechanical force. Note that the equations highlight the fact that the generated electrical energy, (*i.e.*, E = ½QV), is proportional to the product of piezoelectric charge and voltage coefficients.

The key benefit of the piezoelectric bimorph is that this structure makes the resonant frequency low, allowing the cantilever system to match the frequency constant that can be generated by ambient vibrations, such as the unsteady flow. A piezoelectric harvester generates the maximum power when it is made to vibrate at its resonant frequency. Thus, the important requirement for the harvester design is to match the frequency constant of the unsteady flow to the resonance frequency of the vibrating structure. Recent investigations of flow energy piezoelectric harvesters showed that the piezoelectric bimorphs with optimum nozzle configuration (a spline nozzle design) produced >3 mW due to matched resonant frequency of the unsteady flow, promising for high temperature energy harvesters [101].

The major drawback of a piezoelectric bimorph that consists of monolithic piezoelectric layers is its brittle nature. It was shown that when driven at high flow rates, the strain in the bimorph exceeded the ultimate strain and formed cracks, causing a short life-time as well as output power degradation. This problem can be overcome using piezocomposite layers. The idea of using piezoelectric composites to overcome the brittleness of piezoelectric ceramics has been reported, demonstrating improved lifetime and reliability over standard monolithic piezoelectric ceramics. For example, the active fiber composite (AFC) or macro fiber composite (MFC) offer high flexibility and extreme durability compared to monolithic piezoelectric materials. The reliability and life cycle test results of this type of composites, such as MFC, demonstrated that the composites was capable of producing a peak-to-peak actuation strain of approximately 2000 parts-per-million in the longitudinal direction, with no degradation up to 100 million electrical cycles, and no net reductions in actuation amplitude at elevated temperatures up to 65 °C, which is the maximum operational temperature of the polymer used [58]. Note that the maximum operating temperature of composites can be further increased with the selection of high temperature polymer materials, promising for reliable, long lifetime, high temperature energy harvesting applications.

## 5. Conclusions

This paper has reviewed research progress on high power and high temperature transducers and actuators using piezocomposites. Piezoelectric composites offer several advantages over monolithic piezoelectrics. A large voltage coefficient of piezocomposites combined with a large charge coefficient increases the sensitivity of the devices, making them attractive for sensors, transducers and energy harvesting applications. The characteristic of low lateral mode interference in piezocomposites is particularly interesting for ultrasonic wire-bonding, improving the axial vibration and bond quality. For the case of therapeutic ultrasound, high mechanical flexibility and low lateral mode interference improves focusing capability. The benefits of piezocomposites are not only the improvement of the electromechanical properties but also the durability and reliability as a result of flexible nature, greater robustness and longer life-time of the piezoelectric devices.

Despite their promise, high power and high temperature piezocomposites still require research efforts. Challenges associated with high temperature and high power composites are availability of the proper passive filler materials. There were several studies that showed promise for use of composites in high temperature and high power applications, with use of glass sphere modified epoxies and low loss epoxies, respectively. With the advance of new material development, it is anticipated that high temperature and high power piezocomposites will become a more mature technology, and it is likely that the next few years will see an increase in the number of high temperature and/or high power piezocomposite transducers.

## Figures and Tables

**Table 1.** The dielectric, piezoelectric and mechanical properties of selected piezoelectric materials for high power and high temperature applications. (KNN: (K_0.5_Na_0.5_)NbO_3_, KCN: K_4_CuNb_8_O_23_, BNT: Bi_0.5_Na_0.5_TiO_3_, BKT: Bi_0.5_K_0.5_TiO_3_, BT: BaTiO_3_).

**Material**		**T_m_/T_C_ (°C)**		**Structure**		**Dielectric Properties**	**Coupling Factors**	**Piezoelectric Coefficients (pC/N)**			**Mechanical Properties**			**Ref.**
High power piezoelectric materials														

PZT4		328		Perovskite		K_33_^T^ ∼ 1300, tanδ ∼ 0.4%	k_t_ ∼ 0.51, k_31_ ∼ −0.33, k_33_ ∼ 0.70		d_33_ ∼ 289, d_15_ ∼ −126				ρ = 7.9g/cc, Q_m_ = 1000	[17]

PZT8		300		Perovskite		K_33_^T^ ∼ 1000, tanδ ∼ 0.4%	k_t_ ∼ 0.48, k_31_ ∼ −0.3, k_33_ ∼ 0.64		d_33_ ∼ 225, d_15_ ∼ −97				ρ = 7.9g/cc, Q_m_ = 1000	[17]

BiScO_3_-PbTiO_3_-Mn		442		Perovskite		K_33_^T^ ∼ 1450, tanδ ∼ 1%	k_t_ ∼ 0.49, k_31_ ∼ −0.33, k_33_ ∼ 0.69		d_33_ ∼ 360, d_15_ ∼ 520				ρ = 7.7g/cc, Q_m_ = 100	[18]

BaTiO_3_-CaTiO_3_-Co		105		Perovskite		K_33_^T^ ∼ 1420, tanδ ∼ 0.5%	k_p_ ∼ 0.31, k_33_ ∼ 0.46		d_33_ ∼ 150				ρ = 6.02g/cc, Q_m_ = 800	[19]

KNN-KCN		T_OT_∼190		Perovskite		K_33_^T^ ∼ 290, tanδ ∼ 0.6%	k_p_ ∼ 0.36, k_31_ ∼ 0.21, k_33_ ∼ 0.55		d_33_ ∼ 90, d_15_ ∼ 125				ρ = 4.4g/cc, Q_m_ = 1500	[20]

BNT-BKT-BT-Mn		T_d_ ∼ 232		Perovskite		K_33_^T^ ∼ 510, tanδ ∼ 0.6%	k_p_ ∼ 0.12, k_31_ ∼ 0.07, k_33_ ∼ 0.46		d_33_ ∼ 96, d_15_ ∼ 153				ρ = 5.8g/cc, Q_m_ = 1100	[10]

<001> Mn:PINMNT		T_d_ ∼ 123		Perovskite		K_33_^T^ ∼ 3600, tanδ ∼ 0.4%	k_33_ ∼ 0.90		d_33_ ∼ 1200				ρ = 8.1g/cc, Q_m_ = 800	[12,21]

<110> Mn:PINMNT		T_d_ ∼ 123		Perovskite		K_33_^T^ ∼ 3000, tanδ ∼ 0.4%	k_33_ ∼ 0.89		d_33_ ∼ 900				ρ = 8.1g/cc, Q_m_ = 1050	[12]

High temperature piezoelectric materials														

PZT5A *	365		Perovskite		K_33_^T^ ∼ 1700, tanδ ∼ 2%		k_t_ ∼ 0.49, k_31_ ∼ −0.34, k_33_ ∼ 0.71			d_33_ ∼ 370, d_31_ ∼ −171		ρ = 7.9g/cc, Q_m_ = 75		[17]

PbTiO_3_	400		Perovskite		K_33_^T^ ∼ 210, tanδ ∼ 1.4%		k_t_ ∼ 0.4, k_31_ ∼ 0.05, k_33_ ∼ 0.40			d_33_ ∼ 50, d_15_ ∼ 40		Q_m_ > 500		[22]

BiScO_3_-PbTiO_3_	450		Perovskite		K_33_^T^ ∼ 2010, tanδ ∼ 5%		k_t_ ∼ 0.49, k_31_ ∼ −0.22, k_33_ ∼ 0.69			d_33_ ∼ 401, d_15_ ∼ 520		ρ = 7.7g/cc, Q_m_ = 50		[23]

Lead metaniobate	500		Tungsten Bronze		K_33_^T^ ∼ 220, tanδ ∼ 0.6%		k_t_ ∼ 0.34			d_33_ ∼ 100, d_15_ ∼ 50		ρ = 5.6 g/cc, Q_m_ = 15–25		[22]

Bi_4_Ti_3_O_12_	650		Bismuth layer		K_33_^T^ ∼ 120, tanδ ∼ 0.4%		k_t_ ∼ 0.2, k_31_ ∼ 0.02, k_33_ ∼ 0.09			d_33_ ∼ 18, d_15_ ∼ 16		ρ = 6.55 g/cc, s_33_^E^∼44 pm^2^/N, Q_m_ > 600		[22]

LiNbO_3_	1150		Corundum		K_33_^T^ ∼ 25, tanδ ∼ 0.5%		k_t_ ∼ 0.17 (z cut), 0.49 (y/36° cut)			d_31_ = −1, d_33_ = 6, d_15_ = 68, d_22_ = 21		ρ = 4.65 g/cc, Q_m_ = 10,000		[24]

* Note: Modified PZT5A materials possess high Curie temperatures >380 °C that can be used for high temperature (200–250 °C) applications [25].

**Table 2.** Various properties of passive polymer materials. ρ, Y, σ, k_c_, T_g_, and T_m_ are density, Young's modulus, Poisson's ratio, thermal conductivity, glass transition temperature, and maximum working temperature, respectively. α_L_ and α_S_ and longitudinal and shear attenuation coefficients, respectively.

**Material**	**Filler**	**ρ (g/cm^3^)**	**Y (GPa)**	**σ**	**k_c_ (W/m.K)**	**T_g_ (°C)**	**α_L_/α_S_ (dB/m/MHz)**	**Ref.**
Low temperature (<100 °C) resins								

Epotek 301		1150	3.6	0.35	0.18	80	-	[27]
Spurr		1135	3.01	0.37	0.3	-	236/-	[28]
CY1301/HY1300		1149	4.28	0.36	0.22	60	287/738	[29–31]
CY221/HY956 (5:1)		1134	3.53	0.37	-	40	895/4108	[30,32]
HYSOL 2038/3404		1150	5.28	0.38	-	55	-	[33,34]
STYCAST 2651-40	Mica	1503	8.88	0.32	0.6	87	352/726	[29–31]
STYCAST 2850-FT	Al_2_O_3_	2292	18.99	0.31	1.36	84	269/599	[29–31]
T7110		2164	12.4	0.25	1	60	410/-	[28]

High temperature (>100 °C) resins								

EP3512		1328	2.82	0.41	0.2	300 (T_m_)	105/-	[28]
STYCAST W67		1210	4.46	0.34	0.38	230 (T_m_)	269/599	[32,35]
MY750/HY906/DY062	Al_2_O_3_	2007	11.3	0.31	0.38	151 (T_g_)	348/669	[29–31]
Duralco 4538		1092	1.46	0.44	-	232 (T_m_)	973/8808	[32,36]
Duralco 128		1610	9.01	0.31	4.5	232 (T_m_)	577	[28,36]

**Table 3.** Comparison of the material properties for piezoelectric ceramics and composites. (Positive and negative sign indicates advantages and disadvantages for ultrasound transducer applications, respectively.)

**Parameter**	**Ceramic**	**Composites**
Coupling factor	High (+)	High (+)
Acoustic impedance	High (-)	Low (+)
Permittivity	High (+)	Medium (+)
Spurious modes	Many (-)	Weak (+)
Flexibility	Stiff (-)	Flexible (+)
